# Sex differences in factors influencing hospital-acquired pneumonia in schizophrenia patients receiving modified electroconvulsive therapy

**DOI:** 10.3389/fpsyt.2023.1127262

**Published:** 2023-02-14

**Authors:** Mi Yang, Yan Yang, Liju Liu, Di Kong, Min Xu, Xincheng Huang, Cheng Luo, Guocheng Zhao, Xiangyang Zhang, Yan Huang, Yunzhong Tu, Zezhi Li

**Affiliations:** ^1^Department of Psychiatry, The Fourth People's Hospital of Chengdu, Chengdu, China; ^2^The Clinical Hospital of Chengdu Brain Science Institute, MOE Key Lab for Neuroinformation, University of Electronic Science and Technology of China, Chengdu, China; ^3^School of Life Science and Technology, University of Electronic Science and Technology of China, Chengdu, China; ^4^CAS Key Laboratory of Mental Health, Institute of Psychology, Chinese Academy of Sciences, Beijing, China; ^5^Department of Psychiatry, Chongqing Mental Health Center, Chongqing, China; ^6^Department of Psychiatry, The Affiliated Brain Hospital of Guangzhou Medical University, Guangzhou, China; ^7^Department of Psychiatry, Guangdong Engineering Technology Research Center for Translational Medicine of Mental Disorders, Guangzhou, China

**Keywords:** sex difference, modified electroconvulsive therapy, schizophrenia, hospital-acquired pneumonia, non-antipsychotics

## Abstract

**Background:**

Sex differences may be presented in the clinical features or symptoms of schizophrenia patients but also affect the occurrence of hospital-acquired pneumonia (HAP). Modified electroconvulsive therapy (mECT) is a common treatment method for schizophrenia, used in combination with antipsychotics. This retrospective research explores the sex difference in HAP affecting patients with schizophrenia who have received mECT treatment during hospitalization.

**Methods:**

We included schizophrenia inpatients treated with mECT and antipsychotics between January 2015 and April 2022. Blood-related and demographic data collected on admission were analyzed. Influencing factors of HAP in male and female groups were assessed separately.

**Results:**

A total of 951 schizophrenia patients treated with mECT were enrolled in the study, including 375 males and 576 females, of which 62 patients experienced HAP during hospitalization. The risk period of HAP in these patients was found to be the first day after each mECT treatment and the first three sessions of mECT treatment. Statistically significant differences in the incidence of HAP were identified in male vs. female groups, with an incidence in men about 2.3 times higher than that in women (*P* < 0.001). Lower total cholesterol (*Z* = −2.147, *P* = 0.032) and the use of anti-parkinsonian drugs (χ^2^ = 17.973, *P* < 0.001) were found to be independent risk factors of HAP in male patients, while lower lymphocyte count (*Z* = −2.408, *P* = 0.016), hypertension (χ^2^ = 9.096, *P* = 0.003), and use of sedative-hypnotic drugs (χ^2^ = 13.636, *P* < 0.001) were identified in female patients.

**Conclusion:**

Influencing factors of HAP in schizophrenia patients treated with mECT have gender differences. The first day after each mECT treatment and the first three sessions of mECT treatment were identified to have the greatest risk for HAP development. Therefore, it would be imperative to monitor clinical management and medications during this period according to these gender differences.

## 1. Introduction

Schizophrenia (SCZ) is a common severe psychiatric disorder and usually manifests clinically with psychotic symptoms such as hallucinations, delusions, emotional indifference, and cognitive dysfunction (1, 2). According to a 2018 World Health Organization report, more than 20 million people are already living with SCZ worldwide, and China accounts for about half of this population (3). SCZ is characterized by high incidence, high disability, and a low cure rate, with a lifetime incidence of about 1% (4). As such, the disease brings heavy psychological and economic burdens to patient families and broader society (5, 6) and has become a major societal challenge (4).

Gender differences may exist in the clinical symptoms of schizophrenia patients; for example, the age of onset for men may be a 3-year younger than that for women (7, 8). The correlation between non-social functioning and objective social cognition in men may be much stronger than in women (9), whereas hostile bias correlates with verbal fluency found in women (10). Women are good at processing speed and verbal situational memory, but men are good at visual working memory (11). There are also sex differences in the cognitive correlates of first-episode schizophrenia (12), i.e., working memory and executive function were correlated to onset age, negative symptoms were associated with memory or working memory in women, whereas processing speed was correlated with antipsychotic dosage in men. Antipsychotics may induce gender differences in extrapyramidal and anticholinergic responses, sexual problems, and subjective tolerance (13). However, no sex differences in the efficacy of amisulpride or risperidone medication were found in elderly schizophrenia patients (14). Although women may be more prone to gain weight from the antipsychotic medication (15), women may have a better prognosis than men (16). According to the current pathology of schizophrenia, these gender differences may be related to gene expression (17), the mechanism of the microbiota-brain-gut axis (18), differences in brain structure and function (19), or even sociocultural (20). However, these hypotheses are inconsistent to a large extent, so the mechanisms underlying the clinical characterization induced by gender differences need further investigation.

Electroconvulsive therapy (ECT) is widely used, particularly in patients with refractory schizophrenia (21–24). To avoid the generalized convulsions triggered by ECT treatment and the related fear of convulsions (25), modified ECT (mECT) was developed. Due to the use of anesthetic and muscle relaxants before the mECT treatment (26, 27), the risk of post-treatment infections may be increased (28). Prior research on ECT has shown that the incidence of pneumonia infection is 3.8 per 10,000 ECT treatments (29). Hospital-acquired pneumonia (HAP) is a leading cause of morbidity and mortality, and the incidence of HAP remains high, but effective treatment is usually lacking (30). Sex differences in the incidence of HAP exist (31), with men being a risk factor for HAP (32, 33), but a lower incidence of non-ventilator HAP in women (34). Some pathologies may be explained by these differences induced by genders, such as immune response to the virus (24), diabetes (35), or chronic obstructive pulmonary disease (COPD) (36). However, there are few studies on gender differences in risk factors related to hospital-acquired pneumonia (HAP) in SCZ patients with mECT around the world. Therefore, this paper aims to analyze the risk factors associated with the development of HAP in SCZ patients who have received mECT in recent years, explore the possible pathogenesis of HAP caused by gender differences, and provide a basis for guiding clinical management and improving the quality of patient treatment.

## 2. Materials and methods

### 2.1. Patients

This retrospective study included inpatients with schizophrenia admitted between January 2015 and April 2022. Patients met the diagnosis criteria of schizophrenia according to the *International Classification of Diseases*-10 (*ICD*-10) and received mECT treatment during their hospitalization. The diagnosis of HAP required all the following criteria: new lung infiltrates on chest imaging, respiratory decline, fever, and productive cough (37). Patients with infections within 48 h of hospitalization were excluded. This study was approved by the Ethics Committee of the Fourth People's Hospital of Chengdu.

Patient information collected included name, age, gender, as well as the status of diabetes mellitus, hypertension, epilepsy, or substance dependence (smoking or drinking), and excluded patients with comorbid cardiovascular disease. Blood samples were collected on admission for routine biochemical testing (white blood cells, red blood cells, platelets, lipids, glucose, blood proteins, etc.). Other medications of patients receiving mECT at the time of hospitalization were recorded, such as sedative-hypnotic drugs (SHD), antidepressant drugs (ADD), anti-anxiety drugs (AAD), antimanic drugs (AMD), anti-epileptic drugs (AED), anti-parkinsonian drugs (APD), and other neurological drugs. mECT-related conditions only for patients with HAP, such as the days from the first mECT to HAP (dfE2H) occurrence, the numbers of mECT treatments before HAP (nE2H) occurrence, and the days from the last mECT treatment to HAP (dlE2H) occurrence.

### 2.2. mECT parameters

The ECT Instrument was Thymatron System IV (Somatics, LLC, 149 Amityville Street Islip Terrace, NY 11752 USA). Electrode placement was a bilateral temporal energization mode. The electrical stimulation procedure was LOW 0.5: Frequency: 10–70 Hz; Pulse width: 0.5 ms; Duration: ≤ 8s; Waveform: bipolar, brief pulsed, square wave. Stimulus intensity (power): “Age mode” is used. For the first treatment session, the power is set to age × 80% for younger than 30 and age × 100% for older than 50; The power of follow-up treatment increased by 1–5% depending on the seizure index. Convulsions quality assessments: EEG Endpoint was 20–60 s; Average Seizure Energy Index was over 5,000; Postictal Suppression Index was over 80%.

### 2.3. Statistical analysis

Software SPSS 26 (*IBM Corporation, New Orchard Road, Armonk, NY 10504, USA*) was used for statistical calculations. These patients were divided into two groups by gender, with subgroups of HAP and non-HAP. Then the statistical analysis was conducted as follows: First, a general linear model (univariate model) was used for assessing the interaction effect of gender vs. other factors on HAP. Second, influencing factors for HAP men and women were analyzed separately. The χ^2^ test was used for categorical variables. Continuous variables were first tested for normality by *Kolmogorov-Smirnov*; if variables conformed to a normal distribution, *t-*testing was used, while non-normality variables were tested by non-parametric (*Mann-Whitney*) test. Since the included data were almost all non-normal variables, *Spearman* correlation analysis was used. Third, binary logistic regression modeling was later used for risk factors analysis. Finally, statistical calibration was performed using the *Bonferroni* method (*P* < 0.05/31 ≈ 0.0016). Continuous variables were expressed as mean ± standard deviation (*x̄* ± *std*.), and *P* < 0.05 was considered to be statistically significant.

## 3. Results

### 3.1. General characteristics of included SCZ patients

A total of 951 inpatients, aged between 14 and 71 years old, were included. Of the 951 inpatients, 375 were male, and 576 were female, with mean ages of 32.57 ± 11.93 years and 36.85 ± 13.96 years, respectively, and males being significantly younger than females (*t* = 5.053, *P* < 0.001). 62 inpatients were HAP, with 37 in men and 25 in women. Covariance analysis results indicate interaction between sex and epilepsy, TC, anti-depressant drugs, anti-epileptic drugs, anti-parkinsonian drugs, and other neuron drugs (all *P*s < 0.05), as shown in Table 1. Only epilepsy passed the Bonferroni test.

**Table 1:** Interaction effect between influencing factors and sex on hospital-acquired pneumonia (HAP).

	**Factors**		**Sex**		**Factor** × **Sex**	
	***F***	***P***	***F***	***P***	***F***	***P***
**Demographic data**						
Age (years)	0.106	0.745	2.716	0.100	0.222	0.637
Days in hospital	4.071	0.044	0.688	0.407	1.478	0.224
Hypertension	7.561	0.006	11.763	0.001	0.107	0.744
Diabetes	5.940	0.015	11.589	0.001	0.007	0.935
Epilepsy	8.247	0.004	12.557	<0.001	12.496	<0.001
Substance dependence	0.016	0.899	10.840	0.001	0.313	0.576
**Blood data**						
TB (μmol/L)	1.145	0.285	0.842	0.359	0.628	0.428
DB (μmol/L)	0.120	0.914	3.336	0.068	0.09	0.891
IB (μmol/L)	0.035	0.851	0.808	0.369	0.711	0.399
Glucose (mmol/L)	0.963	0.327	0.263	0.608	0.185	0.667
TC (mmol/L)	4.983	0.026	7.460	0.006	4.164	0.042
TG (mmol/L)	0.395	0.530	2.959	0.086	0.001	0.973
HDL (mmol/L)	0.458	0.499	0.028	0.688	0.451	0.502
LDL (mmol/L)	0.351	0.553	8.051	0.005	3.683	0.055
Albumin (g/L)	1.719	0.190	0.287	0.593	0.672	0.413
Uric acid (μmol/L)	0.920	0.338	1.211	0.271	0.002	0.963
Leukocyte (10^9^/L)	2.207	0.138	0.069	0.793	0.632	0.427
Monocyte (10^9^/L)	1.770	0.184	0.288	0.591	3.212	0.073
Lymphocyte (10^9^/L)	2.450	0.118	0.106	0.745	0.800	0.371
Eosinophils (10^9^/L)	2.439	0.119	7.823	0.005	0.166	0.683
Basophils (10^9^/L)	2.153	0.143	2.721	0.099	0.013	0.909
RBC (10^12^/L)	2.343	0.126	0.244	0.622	0.003	0.960
Hemoglobin (g/L)	0.555	0.456	0.068	0.794	0.014	0.907
Thrombocyte (10^9^/L)	2.552	0.110	3.416	0.065	0.951	0.330
**Affiliated medication**						
Sedative-hypnotics	26.827	<0.001	2.559	0.110	1.641	0.201
Anti-anxiety drugs	0.323	0.570	10.858	0.001	0.046	0.831
Anti-manic drugs	4.374	0.037	10.525	0.001	0.051	0.822
Anti-depressant drugs	2.984	0.084	6.244	0.013	6.422	0.011
Anti-epileptic drugs	7.032	0.008	4.255	0.040	4.191	0.041
Anti-parkinsonian drugs	31.591	<0.001	2.364	0.124	6.444	0.011
Other neuron drugs	6.169	0.013	9.439	0.002	9.552	0.002

DB, direct bilirubin; HDL, high-density lipoprotein; IB, indirect bilirubin; LDL, low-density lipoprotein; RBC, red blood cells; TB, total bilirubin; TC, total cholesterols; TG, triglycerides.

### 3.2. HAP occurrence in patients receiving mECT

HAP occurred in 62 of the 951 study subjects, with an incidence of 6.52%. Patients developed HAP 20.10 ± 18.29 days after admission, received about 3.47 ± 2.57 sessions of mECT treatment before HAP, and HAP occurred approximately 2.78 ± 4.70 days after the last MECT treatment. The prevalence of HAP was significantly higher in men than in women (37/375:25/576 = 9.87%:4.34% ≈ 2.3, χ^2^ = 11.382, *P* < 0.001). There was no statistically significant difference between men and women in dfE2H, nE2H, or dlE2H, as shown in Table 2.

**Table 2:** Modified electroconvulsive therapy (mECT) related information between woman and man.

	**Woman (*****n*** = **25)**		**Man (*****n*** = **37)**		***Z***	***P***
	***x̄*** ± *std*	**Median (Q1, Q3)**	***x̄*** ± *std*	**Median (Q1, Q3)**		
dfE2H	19.39 ± 16.90	13.20 (5.91, 26.38)	20.59 ± 19.38	13.81 (4.79, 32.11)	−0.194	0.846
nE2H	3.45 ± 4.98	1.00 (0.41, 5.52)	2.33 ± 4.51	1.00 (0.39, 2.03)	−0.854	0.393
dlE2H	3.44 ± 2.66	2.00 (2.00, 4.50)	3.49 ± 2.53	3.00 (1.00, 5.50)	−0.037	0.971

dfE2H, days from the first mECT to hospital-acquired pneumonia (HAP) occurrence; nE2H, numbers of mECT treatments before HAP occurrence; dlE2H, days from the last mECT treatment to HAP occurrence.

Q1, Q3 indicate the first and third quartiles.

*x̄* ± *std* Indicates mean and standard deviation. Z and P are calculated by the Mann-Whitney method.

### 3.3. Risk factors for HAP in male SCZ patients receiving mECT

In Table 3, hospitalized days were significantly higher in the HAP group, as compared to the non-HAP group (*Z* = −2.760, *P* = 0.006). Total cholesterol (mmol/L) was significantly lower in the HAP group as compared to the non-HAP group (*Z* = −2.147, *P* = 0.032), while monocyte count was significantly higher (*Z* = −2.001, *P* = 0.045). There was a statistically significant difference in basophils between the HAP and non-HAP group (*Z* = −2.065, *P* = 0.039). There were also statistically significant increases in HAP incidence found in patients who had taken any medications of SHD (χ^2^ = 11.396, *P* < 0.001), ADD (χ^2^ = 5.237, *P* = 0.002), AED (χ^2^ = 7.993, *P* = 0.005), APD (χ^2^ = 17.973, *P* < 0.001), or other neurological drugs (χ^2^ = 7.323, *P* = 0.007), though only SHD and APD passed the Bonferroni test.

**Table 3:** Differences between HAP and non-HAP groups in male SCZ patients with mECT.

	**Non-HAP** **(*n* = 338)**	**HAP** **(*n* = 37)**	***t, Z, χ^2^***	***P***
**Demographic data**				
Age (year)	32.58 ± 11.62	32.43 ± 14.66	−0.803	0.422
**Diabetes**				
No	331	35	1.583	0.208
Yes	7	2		
**Hypertension**				
No	331	35	1.583	0.208
Yes	7	2		
**Epilepsy**				
No	336	37	0.220	0.639
Yes	1	0		
**Substance addiction**				
No	333	36	0.317	0.573
Yes	5	1		
Days in hospital (day)	46.51 ± 30.59	55.18 ± 23.85	−2.760	0.006
**Blood parameters**				
TB (μmol/L)	17.00 ± 9.00	16.76 ± 7.95	−0.212	0.832
DB (μmol/L)	4.24 ± 2.66	4.17 ± 2.42	−0.133	0.894
IB (μmol/L)	12.47 ± 6.9	12.91 ± 6.19	−0.736	0.461
Glucose (mmol/L)	4.95 ± 1.27	5.1 ± 1.57	−0.998	0.318
TC (mmol/L)	4.41 ± 1.05	4.00 ± 1.14	−2.147	0.032
TG (mmol/L)	1.22 ± 0.73	1.17 ± 0.66	−0.545	0.586
HDL (mmol/L)	1.38 ± 0.33	1.42 ± 0.38	−0.695	0.554
LDL (mmol/L)	2.27 ± 0.72	2.11 ± 0.54	−1.223	0.221
Albumin (g/L)	42.97 ± 3.71	43.67 ± 3.33	−1.089	0.276
UA (μmol/L)	414.31 ± 131.1	401.62 ± 136.6	−0.443	0.658
Leukocyte (10^9^/L)	7.69 ± 2.47	8.23 ± 3.05	−0.814	0.416
Monocyte (10^9^/L)	0.49 ± 0.18	0.54 ± 0.16	−2.001	0.045
Lymphocyte (10^9^/L)	1.77 ± 0.65	1.73 ± 0.54	−0.093	0.926
Eosinophils (10^9^/L)	0.12 ± 0.11	0.10 ± 0.10	−1.012	0.311
Basophils (10^9^/L)	0.03 ± 0.02	0.03 ± 0.03	−2.065	0.039
RBC (10^12^/L)	4.88 ± 0.57	4.79 ± 0.36	−0.660	0.509
Hemoglobin (g/L)	146.37 ± 13.71	145.57 ± 12.87	−0.218	0.827
Thrombocyte (10^9^/L)	209.41 ± 62.92	194.81 ± 70.01	−1.187	0.235
**Medications Sedative-hypnotics**				
No	181	9	11.396	<0.001
Yes	157	28		
**Anti-anxiety**				
No	337	37	0.110	0.740
Yes	1	0		
**Anti-manic**				
No	332	35	2.105	0.147
Yes	6	2		
**Antidepressants**				
No	312	30	5.237	0.002
Yes	26	7		
**Anti-epileptics**				
No	283	24	7.993	0.005
Yes	55	13		
**Anti-Parkinsonians**				
No	250	15	17.973	<0.001
Yes	88	22		
**Other neuron drugs**				
No	336	35	7.323	0.007
Yes	2	2		

DB, direct bilirubin; HAP, hospital-acquired pneumonia; HDL, high-density lipoprotein; IB, indirect bilirubin; LDL, low-density lipoprotein; RBC, red blood cells; TB, total bilirubin; TC, total cholesterols; TG, triglycerides.

There were correlations between HAP and days in hospital (r_s_ = 0.143, *P* = 0.006), total cholesterol (r_s_ = −0.111, *P* = 0.032), monocyte count (r_s_ = 0.103, *P* = 0.045), SHD (r_s_ = 0.174, *P* = 0.001), ADD (r_s_ = 0.118, *P* = 0.022), AED (r_s_ = 0.146, *P* = 0.005), APD (r_s_ = 0.219, *P* < 0.001), and other neurological drugs (r_s_ = 0.140, *P* = 0.007) by Spearman test. After input of these variables into the logistic regression equation, only total cholesterol [Beta = −0.373, Wald = 3.920, *P* = 0.048, Exp(B) = 0.688 (0.476, 0.996)] and APD [Beta = 1.366, Wald = 14.84, *P* < 0.001, Exp(B) = 3.920 (1.925, 7.981)] survived.

### 3.4. Risk factors for HAP in female SCZ patients receiving mECT

In Table 4, statistically significant higher incidences of diabetes (χ^2^ = 6.203, *P* = 0.013), hypertension (χ^2^ = 9.096, *P* = 0.003), epilepsy (χ^2^ = 5.643, *P* < 0.001), lymphocyte count (Z = −2.408, *P* = 0.016), eosinophil count (*Z* = −2.141, *P* = *0*.032), SHD (χ^2^ = 13.636, *P* < 0.001), and APD (χ^2^ = 8.526, *P* = 0.004) were identified in the HAP group, as compared to the non-HAP group, while lower levels of total bilirubin (*Z* = −2.024, *P* = *0*.043) on admission were found in the HAP group. Only variables of epilepsy and SHD passed the *Bonferroni* test.

**Table 4:** Differences between HAP and non-HAP groups in female SCZ patients with mECT.

	**Non-HAP** **(*n* = 551)**	**HAP** **(*n* = 25)**	***t, Z, χ^2^***	***P***
**Demographic data**				
Age (year)	36.74 ± 13.80	39.16 ± 17.22	−0.508	0.611
**Diabetes**				
No	535	22	6.203	0.013
Yes	16	3		
**Hypertension**				
No	539	22	9.096	0.003
Yes	12	3		
**Epilepsy**				
No	551	24	22.078	<0.001
Yes	0	1		
**Substance addiction**				
No	549	25	0.091	0.763
Yes	2	0		
Days in hospital (day)	46.43 ± 32.16	51.67 ± 30.44	−1.301	0.193
**Blood parameters**				
TB (μmol/L)	15.39 ± 7.92	12.70 ± 7.18	−2.024	0.043
DB (μmol/L)	3.24 ± 1.68	3.25 ± 2.13	−0.774	0.439
IB (μmol/L)	10.69 ± 5.80	9.62 ± 5.77	−0.923	0.356
Glucose (mmol/L)	5.25 ± 1.64	5.45 ± 1.31	−1.110	0.267
TC (mmol/L)	4.58 ± 1.06	4.54 ± 1.00	−0.176	0.861
TG (mmol/L)	1.11 ± 0.58	1.05 ± 0.54	−0.621	0.535
HDL (mmol/L)	1.57 ± 0.37	1.57 ± 0.40	−0.088	0.930
LDL (mmol/L)	2.26 ± 0.69	2.45 ± 0.78	−1.079	0.281
Albumin (g/L)	41.54 ± 3.96	41.94 ± 3.91	−0.573	0.567
UA (μmol/L)	312.70 ± 97.62	296.48 ± 74.93	−0.481	0.630
Leukocyte (10^9^/L)	7.29 ± 2.39	7.61 ± 2.54	−1.103	0.270
Monocyte (10^9^/L)	0.43 ± 0.18	0.42 ± 0.15	−0.299	0.765
Lymphocyte (10^9^/L)	1.76 ± 0.63	1.46 ± 0.56	−2.408	0.016
Eosinophils (10^9^/L)	0.11 ± 0.16	0.06 ± 0.06	−2.141	0.032
Basophils (10^9^/L)	0.03 ± 0.02	0.02 ± 0.02	−1.640	0.101
RBC (10^12^/L)	4.32 ± 0.47	4.22 ± 0.47	−1.030	0.303
Hemoglobin (g/L)	127.02 ± 12.76	124.92 ± 15.12	−0.567	0.571
Thrombocyte (10^9^/L)	230.49 ± 67.93	221.8 ± 75.92	−0.464	0.643
**Medications Sedative-hypnotics**				
No	274	3	13.636	<0.001
Yes	277	22		
**Anti-anxiety**				
No	533	25	0.843	0.359
Yes	18	0		
**Anti-manics**				
No	546	24	2.219	0.136
Yes	5	1		
**Anti-depressants**				
No	506	24	0.565	0.452
Yes	45	1		
**Anti-epileptics**				
No	501	22	0.245	0.621
Yes	50	3		
**Anti-Parkinsonians**				
No	393	11	8.526	0.004
Yes	158	14		
**Other Neuron Drugs**				
No	541	25	0.462	0.497
Yes	10	0		

DB, direct bilirubin; HAP, hospital-acquired pneumonia; HDL, high-density lipoprotein; IB, indirect bilirubin; LDL, low-density lipoprotein; RBC, red blood cells; TB, total bilirubin; TC, total cholesterols; TG, triglycerides.

*Spearman* testing showed that HAP is correlated with diabetes (*r*_s_ = 0.104, *P* = *0*.013), hypertension (*r*_s_ = 0.126, *P* = *0*.003), epilepsy (*r*_s_ = 0.196, *P* < 0.001), total bilirubin (*r*_s_ = −0.084, *P* = 0.043), lymphocyte count (*r*_s_ = −0.100, *P* = *0*.016), eosinophil count (*r*_s_ = −0.089, *P* = 0.032), SHD (*r*_s_ = 0.154, *P* < 0.001), and APD (*r*_s_ = 0.122, *P* = 0.003). After input of these variables into the logistic regression equation, only lymphocyte count [*Beta* = 1.702, *Wald* = 5.432, *P* = 0.020, *Exp*(B) = 5.483 (1.311, 22.937)], hypertension [*Beta* = −0.835, *Wald* = 4.764, *P* = 0.029, *Exp*(B) = 0.434 (0.205, 0.918)], and SHD [*Beta* = 2.287, *Wald* = 9.387, *P* = 0.002, *Exp*(B) = 9.847 (2.280, 42.536)] survived.

## 4. Discussion

To the best of our knowledge, this is the first study to investigate the risk factors of HAP in patients with mECT treatment. We found that the incidence of HAP in mECT patients was 6.52%, greater than the 1.80% previously reported by Han et al. in patients with schizophrenia spectrum disorder (38), but slightly less than the 7.8% reported in a study of elderly SCZ patients (age >50) by Yang et al. (39).

ECT treatment improves the structure and function of the hippocampus and insula in SCZ patients and regulates the function of the prefrontal and thalamic striatum of the default network (40, 41). Animal experiments have also shown that ECT attenuates microglia and astrocyte proliferation, thus improving schizophrenic behavior (42). However, ECT treatment may also induce acute immunoinflammatory responses, such as elevated plasma cortisol and levels of interleukin-1 or−6; lowered levels of blood tumor necrosis factor alpha (TNF-α) and interleukin-6 in long-term treatment (43); or even detrimentally alter blood parameters (44), leading to a decrease in immunity. Schizophrenia itself is also a risk factor for the development of pneumonia (45), thus ECT may increase the risk of HAP in patients, and our prior work (46) had found that mECT may be a risk factor influencing the occurrence of HAP. Further, we wanted to find whether there were gender differences in HAP, and the discussion of the results was divided into the following four parts.

### 4.1. mECT induced increased incidence of HAP

Our results showed that among HAP patients who underwent mECT, there was no statistical difference between men and women in the three indicators: days from first mECT treatment to HAP occurrence (dfE2H), the numbers of mECT treatments before HAP occurrence (nE2H), and days from last mECT treatment to HAP occurrence (dlE2H). However, the results showed that the median number of days from the last mECT treatment to HAP was 1 day in both men and women, indicating that all patients had a very high risk of developing HAP within 1 day after receiving mECT treatment. In addition, the risk of HAP within the first three mECT treatments was quite high in men and women, respectively, which may be due to the fact that patients need to gradually adapt to the clinical symptoms that may occur after mECT and ignore the possible risk of HAP. Therefore, healthcare providers should be on alert the first day after each mECT treatment, and special attention should be paid to clinical care after the first 3 sessions of mECT treatment.

Interestingly, we found that male patients with SCZ are more prone to HAP than women, and the risk factors predisposing both groups to HAP also differ from each other.

### 4.2. Risk factors for male mECT patients

The prevalence of HAP in male mECT patients in this study was significantly higher than that in women (male incidence ~2.3 times that of females). This increased prevalence may be related to lifestyle habits of male patients, such as smoking, alcohol abuse, low weight, frequent contact with children, and poor oral hygiene, which are all risk factors for HAP (47). In addition, this study found that lower total cholesterol prior to hospital admission and APD medication in hospitalization may be risk factors for the development of HAP in men.

Our results showed that male SCZ patients treated with mECT with lower total cholesterol levels at admission were more likely to develop HAP, and logistic regression analysis showed that low levels of total cholesterol might be an independent risk factor for HAP in SCZ patients treated with mECT. Cholesterol is widely distributed in many tissues of the body, especially the brain and neural tissues, and may be associated with a variety of diseases (48), and even mental status or personality changes (49). Importantly, cholesterol has an important role in coronavirus entry, membrane fusion, and pathological syncytium formation. 25-hydroxycholesterol (25HC) is one of the metabolites of cholesterol, and 25HC inhibits coronavirus infection by blocking membrane fusion (50), so lower total cholesterol levels may lead to lower 25HC levels, increasing the chance of coronavirus infection in SCZ patients and potentially explaining the correlation between viral infection and lower cholesterol levels (51, 52). Lower total cholesterol may also be a factor of increased short-term mortality in elderly patients with community-acquired pneumonia (CAP) (53). In addition, men may be more sensitive to low levels of cholesterol (54), which accounts for the fact that men with low total cholesterol levels were more likely to develop HAP in our study, whereas women showed no statistical difference in HAP based on the level of total cholesterol.

APDs are used to improve Parkinsonian-like symptoms and extrapyramidal effects in schizophrenic patients. Our results show that male mECT SCZ patients using APDs during hospitalization were more likely to develop HAP, and logistic regression analysis showed that APD use is an independent risk factor for the incidence of HAP. Relatedly, it has been shown that anticholinergic drugs may increase the risk of HAP (55); despite some studies showing that amantadine can be used to prevent pneumonia (56), the presence of drowsiness, falls, and skin problems (57) may in turn lead to an increased risk of developing HAP in patients.

### 4.3. Risk factors for female mECT patients

Risk factors for HAP in women are hypertension, low lymphocyte count on admission, and use of SHDs during hospitalization. There was no statistically significant difference in the prevalence of hypertension between men and women in patients treated with mECT (9/375:15/576, χ^2^ = 0.038, *P* = 0.844), however, SCZ patients with comorbid hypertension in women had a higher risk of HAP, compared to men. ECT treatment may not result in significant changes in blood pressure in hypertensive patients (58), however, patients with comorbidities such as chronic renal insufficiency or diabetes mellitus are more likely to be affected by COVID-19, potentially fatally (59–61). Therefore, for female SCZ patients with comorbid diseases such as hypertension, clinical management should be particularly strengthened during hospitalization to reduce the risk of HAP and improve the quality of patient survival.

Lymphocytes have an important role in immune regulation and can be involved in the pathogenesis of respiratory diseases such as pneumonia, infections, asthma, and acute respiratory distress syndrome (62). The lower lymphocyte count in female mECT patients suggests that immunity may be reduced, potentially increasing the incidence of HAP.

Benzodiazepines drugs (BZD) are often used in sedation-hypnosis and can modulate peripheral γ-aminobutyric acid (GABA) type A on macrophages and increase the incidence of infection by inhibiting proinflammatory cytokines (63). Experiments performed on mice have further shown that benzodiazepines enhance GABA signaling, leading to increased mortality from pneumonia (64). In addition, certain meta-analyses have shown an increased risk of pneumonia with recent or current exposure to benzodiazepines (65, 66), which is consistent with our findings. Therefore, BZDs should be used with caution in SCZ patients receiving mECT.

### 4.4. Limitations

Although mECT is used as a routine treatment for patients with schizophrenia, the number of patients receiving it remains low, which may stem from the fear of electrical stimulation. Since the outbreak of COVID-19, more standardized clinical management and increased awareness of personal protection implemented by medical institutions have led to a decrease in the number of patients with mECT experiencing HAP; therefore, random errors induced by small sample size may have a greater impact on the statistical analysis. Incomplete demographic indicators for some patients, such as height, weight, and education, as well as the lack of cognitive assessment of patients with schizophrenia during data collection, prevented our results from fully reflecting the full picture of patients. Data from an individual psychiatric hospital is another limitation, as regional differences, local culture, economic conditions, and ethnic groups of patients may also influence our results. Therefore, these risk factors deserve to be investigated as a prospective study with a larger patient sample size and collaboration of multiple clinical centers.

## 5. Conclusions

Among schizophrenia patients treated with mECT, men were more likely to develop HAP than women. Schizophrenia patients were at very high risk of developing HAP within the first day after each mECT treatment or in the first three sessions of mECT treatment. Lower levels of total cholesterol and use of anti-parkinsonian drugs were identified as independent risk factors of HAP in male patients, while hypertension, lower lymphocyte count on admission, and use of sedative-hypnotic drugs in hospitalization were identified as independent risk factors in female patients. These gender-based differences may be due to differences in physiological immune function, lifestyle habits, and the complicated nature of schizophrenia itself. Our results may help guide future clinical management and care of patients with SCZ, and help elucidate the potential direction of follow-up studies.

## Data availability statement

The raw data supporting the conclusions of this article will be made available by the authors, without undue reservation.

## Ethics statement

The studies involving human participants were reviewed and approved by the Ethics Committee of the Fourth People's Hospital of Chengdu. Written informed consent to participate in this study was provided by the participants' legal guardian/next of kin.

## Author contributions

MY: conception, writing—original draft preparation, and funding acquisition. YY, DK and MX: data curation. LL: validation. XH: resources. GZ and XZ: writing—review. YH: writing—review and editing. YT: project administration and funding acquisition. ZL: writing—review, supervision, and funding acquisition. All authors contributed to the article and approved the submitted version.
